# Identification of cytokines in benign and malignant thymus tumors: based on Mendelian randomization and proteomics

**DOI:** 10.3389/fendo.2024.1390140

**Published:** 2024-05-17

**Authors:** Kangle Zhu, Jingwei Shi, Rusong Yang, Chu Zhou, Zhengcheng Liu

**Affiliations:** ^1^Nanjing Drum Tower Hospital Clinical College of Nanjing Medical University, Nanjing, Jiangsu, China; ^2^Department of Thoracic Surgery, Nanjing Drum Tower Hospital, Affiliated Hospital of Medical School, Nanjing University, Nanjing, Jiangsu, China

**Keywords:** cytokines, thymus tumor, Mendelian randomization, R language, GWAS

## Abstract

**Objective:**

The aim of this study was to identify potential causal cytokines in thymic malignancies and benign tumors from the FinnGen database using Mendelian randomization (MR).

**Methods:**

In this study, data from genome-wide association studies (GWAS) of 91 cytokines were used as exposure factors, and those of thymic malignant tumors and thymic benign tumors were the outcome variables. Two methods were used to determine the causal relationship between exposure factors and outcome variables: inverse variance weighting (IVW) and MR-Egger regression. Sensitivity analysis was performed using three methods, namely, the heterogeneity test, the pleiotropy test, and the leave-one-out test.

**Results:**

There was a causal relationship between the expression of fibroblast growth factor 5, which is a risk factor for thymic malignant tumors, and thymic malignant tumors. C-C motif chemokine 19 expression, T-cell surface glycoprotein CD5 levels, and interleukin-12 subunit beta levels were causally related to thymic malignant tumors and were protective. Adenosine deaminase levels, interleukin-10 receptor subunit beta expression, tumor necrosis factor (TNF)-related apoptosis-inducing ligand levels, and TNF-related activation-induced cytokine levels showed a causal relationship with thymic benign tumors, which are its risk factors. Caspase 8 levels, C-C motif chemokine 28 levels, interleukin-12 subunit beta levels, latency-associated peptide transforming growth factor beta 1 levels, and programmed cell death 1 ligand 1 expression showed a causal relationship with thymic benign tumors, which are protective factors. Sensitivity analysis showed no heterogeneity.

**Conclusion:**

Cytokines showed a causal relationship with benign and malignant thymic tumors. Interleukin-12 subunit beta is a common cytokine that affects malignant and benign thymic tumors.

## 1 Introduction

Thymoma is the most common tumor type of the anterior mediastinum. The latest data show that thymic tumor incidence in China is 4.09/1 million, higher than in other countries worldwide (1). Thymoma is a tumor of the thymic tissue. Thymic epithelial cell-derived tumors are the most common among them. Thymoma accounts for approximately 95% of all thymic tumors (2, 3). Patients with thymoma show complications with various diseases (4), among which myasthenia gravis (MG) is the most common (5, 6). MG involves skeletal muscles of the whole body, and its main clinical characteristics are the volatility of aggravation after exercise and reduction after rest. Based on the pathological classification by the World Health Organization (WHO) (7), thymoma is divided into type A, AB mixed type, and type B. Type B includes B1, B2, and B3. Thymoma can be divided into low- and high-risk types based on the degree of risk. The low-risk type mainly includes types A, AB, and B1; the high-risk type includes B2 and B3. Surgery is the main treatment for thymoma. For resectable Masaoka Koga stages I–III, surgery is often the first choice of treatment (8), whereby complete thymectomy is recommended for the standard surgical procedure. The scope of resection includes complete resection of the thymic tumor, residual thymus, and perithymic adipose tissue (9). If the tumor cannot be completely resected, chemotherapy is required as an adjuvant treatment. However, compared with other thoracic tumors, such as lung cancer (10, 11) and esophageal cancer (12, 13), research on thymoma has progressed slowly, such as the common pathogenesis of combined diseases, the lack of reliable cell lines in the experiment, and the standardized treatment scheme of clinically unresectable thymoma, which merit research.

Cytokines are a class of secreted proteins crucial in intercellular communication and regulation. These proteins regulate biological processes, including immune responses, cell proliferation and differentiation, and inflammatory responses (14). Cytokines can promote or inhibit cellular function and regulate the body’s response to external stimuli to maintain internal homeostasis and combat external threats. Tumor necrosis factor alpha (TNF-α), EGF, and other cytokines promote abnormal proliferation of tumor cells by activating signaling pathways regulating cell proliferation, including MAPK and PI3K/AKT transduction (15, 16). Some cytokines slow down or prevent the self-destruction of tumor cells by inhibiting the apoptotic signaling pathway, such as by suppressing caspase activity (17). Certain cytokines support tumor growth and provide adequate nutrient supply by promoting angiogenesis (neovascularization), including the vascular endothelial growth factor (VEGF) (18, 19). Some cytokines [such as transforming growth factor beta (TGF-β), interleukin-10 (IL-10), and programmed death-ligand 1 (PD-L1)] are characterized by inhibiting immune cell activity and reducing the recognition and attack of tumor cells, thereby facilitating tumor escape from immune system monitoring (20). These complex cytokine interactions constitute the regulatory network of tumorigenesis and development. An in-depth study of the relationship between the role of different cytokines and tumors is expected to provide important guidance for developing more effective anti-tumor treatment strategies.

Mendelian randomization (MR) is a statistical method based on whole-genome sequencing data [genome-wide association studies (GWAS)], which can effectively reduce bias and is used to reveal causality similar to randomized controlled trials (RCTs) (21). MR can be used to evaluate causal inference, using genetic variants as instrumental variables to represent specific exposures, infer causal relationships between exposures and outcomes, and transform phenotype-to-phenotype causal studies into genotype studies. The advantage is that an individual’s genetic variation precedes the outcome of a disease, which eliminates confounding bias due to reverse causality (22). Modern bioinformatics techniques can measure genetic variation with high precision, which largely reduces the estimation bias caused by measurement errors. MR holds considerable importance in thymoma research. Interrogating the causal nexus between the emergence of thymoma and discrete genetic polymorphisms unveils the underlying pathological and physiological underpinnings of thymoma, fostering enhanced comprehension of disease etiology and progression trajectories. Examination of the linkage between genetic variants and thymoma susceptibility elucidates putative targets for pharmaceutical interventions or therapeutic modalities, paving the way for customized pharmacotherapies tailored to specific genetic profiles and augmenting the efficacy of novel drug discovery endeavors (23). By using genotype as a randomization variable, MR can overcome the effects of confounding factors and reverse the causal inference of causality. In this study, we used MR to analyze GWAS data to investigate the causal relationship between cytokine expressions and benign and malignant thymoma.

## 2 Materials and methods

### 2.1 Data sources

GWAS data related to 91 cytokines were used as exposure factors. Single-nucleotide polymorphisms (SNPs) showing significant association with thymic tumors were selected as IVs. GWAS data on thymic malignant tumors and benign tumors were used as outcome variables. Cytokine factor-related GWAS data sources, such as the one provided by Zhao (24), included 91 cytokines (https://www.ebi.ac.uk/gwas/). GWAS data on thymus gland malignant tumor and benign tumor were obtained from https://storage.googleapis.com/finngen-public-data-r10/summary_stats/finngen_R10_C3_THYMUS_EXALLC.gz and https://storage.googleapis.com/finngen-public-data-r10/summary_stats/finngen_R10_CD2_benign_thymir.gz.

Numbers in GWAS data related to thymic malignancies are as follows: C3_THYMUS_EXALLC, sample size (*N* = 314,264), cases (*n* = 71), and controls (*n* = 314,193). Numbers in GWAS data for thymic benign tumors are as follows: CD2_BENIGN_THYMUS, sample size (*N* = 412,181), cases (*n* = 56), and controls (*n* = 412,125) (**Table 1**).

**Table 1:** GWAS data on benign and malignant thymus tumors.

GWAS ID	Trait	Category	Cases	Controls	Number
C3_THYMUS_EXALLC	Malignant neoplasm of thymus	Neoplasms, from cancer register (ICD-O-3)	71	314,193	314,264
CD2_BENIGN_THYMUS	Benign neoplasm: Thymus	Neoplasms from hospital discharges (CD2_)	56	412,125	412,181

### 2.2 Correlation analysis

**Figure 1:**
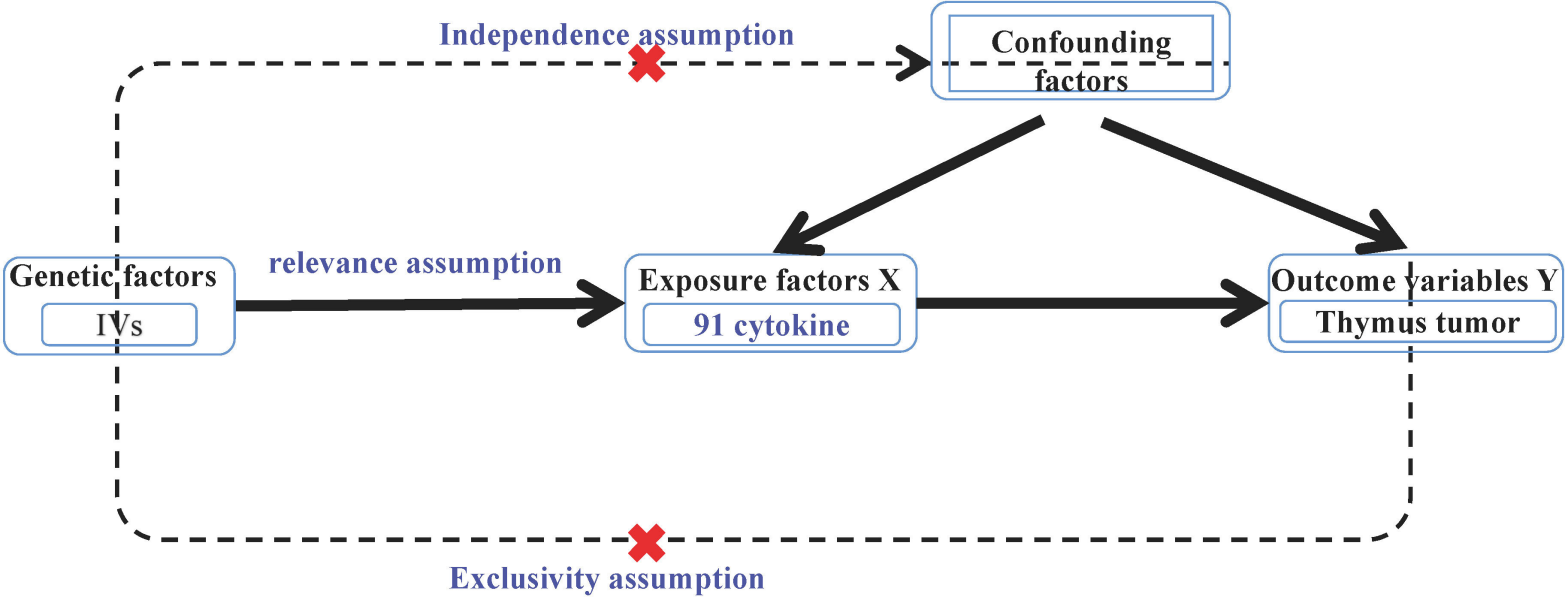
Principle of Mendelian randomization.

MR analysis required the fulfillment of the following three assumptions: ① Association hypothesis: the selected IVs should be strongly associated with benign or malignant thymic tumors (exposure factors). ② Independence hypothesis: the selected IVs, the outcome variables of thymic malignant and benign tumors, and other confounding factors were not associated; and ③ IVs can only affect the weakness of thymic malignant tumors and benign tumors through the effect of cytokines (**Figure 1**) (25).

### 2.3 LDSC score

Linkage disequilibrium (LD) score regression is a statistical method that analyzes the association between genetic variants in genomic data and complex diseases. LD refers to the nonrandom association between different genotypes; the frequency between a pair of genotypes is not equal to the expected frequency of their random combination in the population (26). In genomic data, LD reflects the relative position and association between genes, which is essential for understanding genetic variation and its relationship with disease risk. LD score regression utilizes the information on LD in genomic data to evaluate the association between SNPs and diseases through statistical modeling (27). The rationale is as follows: in the study population, the degree of the LD score between SNPs is an independent variable, while disease status (such as the case–control status) is a dependent variable. SNPs that are significantly associated with disease risk can be identified through regression analysis. Its possible biological mechanism can be explored. Screening criteria were as follows: (1) kb > 10,000; (2) *r*² < 0.001, where kb refers to the regional extent of LD; *r*^2^ ranges from 0 to 1. If *r*²= 1, the two SNPs are in complete LD, and *r*²= 0 suggests complete linkage equilibrium; that is, the assignment of the two SNP_S_ is completely random (28).

### 2.4 Removal of weak IVs

The *F*-statistic assesses the strength of an IV. *F* > 10 is considered a non-weak IV (29). It is calculated as: $F=\frac{N-K-1}{k}\times \frac{R^{2}}{1-R^{2}}$ , where *N* represents the sample size of the exposed data; *K* represents the number of IVs, and *R*^2^ represents the proportion of IVs explaining the exposure. *R*^2^ was calculated as *R*^2^ = 2 × (1 − MAF) × MAF × (β/SD)^2^, where MAF is the minor allele frequency; β is the allele effect size, and SD is the standard deviation (30).

### 2.5 MR analysis

IVW and MR-Egger regression were used to determine the causal relationship between cytokine expression and thymic benign and malignant tumors. The TwoSampleMR package in R was used to visualize the results of MR, including drawing the scatter plot and forest plot. Sensitivity analysis results were plotted. IVW is used to evaluate the reliability of the results of MR analysis, and *p* < 0.05 is defined as a positive result (28). We used the R software (v 4.3.1; https://cloud.r-project.org/) and strawberry perl (5.32.1.1; https://strawberryperl.com/) for MR causality and pleiotropy analyses. *p* < 0.05 denoted a statistically significant result.

### 2.6 Sensitivity analysis

Heterogeneity within the studies was evaluated using the IVW method and the MR-Egger test. *p* < 0.05 indicated the presence of heterogeneity. Heterogeneity represents potential issues within the IVs that extend beyond its direct impact on the exposure factor, suggesting the occurrence of pleiotropy (31). Pleiotropic effects violate the assumptions of independence and exclusivity in MR analysis. The MR-Egger intercept test was conducted to ascertain the presence of pleiotropy and ensure the robustness of the findings. A *p*-value less than 0.05 indicated the presence of pleiotropy within the data. Sensitivity analyses were conducted using the “leave-one-out” method, systematically removing the results of individual SNPs to evaluate their effect on the overall outcomes (32). The outlier status was assessed after each removal, and the stability of the results was assessed. The data were visualized using a funnel plot generated using the R language. The symmetrical distribution of SNPs within the funnel plot was an indicator of reliability. In summary, comprehensive assessment methods, including tests for heterogeneity, pleiotropy, sensitivity analyses, and funnel plot visualization, were utilized to ensure the validity and robustness of the MR analysis (30).

## 3 Results

### 3.1 Screening IVs

**Figure 2:**
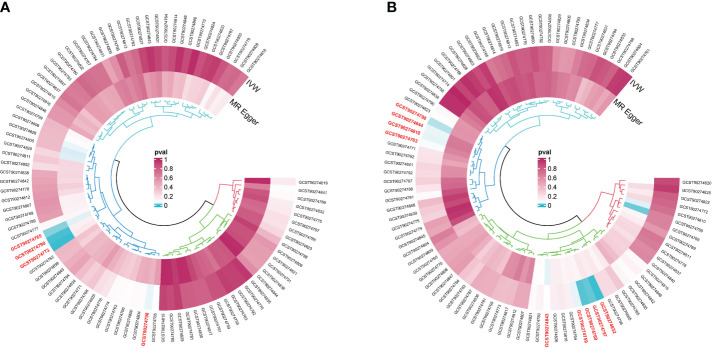
**(A)** GWAS dataset of single-nucleotide polymorphisms (SNPs) associated strongly with cytokine expression and malignant neoplasm of the thymus (controls excluding all cancers). **(B)** GWAS dataset of SNPs associated strongly with cytokines and benign neoplasm: Thymus.

The R packages “VariantAnnotation,” “gwasglue,” “dplyr,” and “tidyr” were used to select SNPs exhibiting a strong correlation between exposure factors and outcome factors. The filtering criterion was *p* < 5e-05. Four SNP datasets showed strong associations with thymic malignancies, namely, C-C motif chemokine 19 levels (GCST90274765), T-cell surface glycoprotein CD5 levels (GCST90274773), fibroblast growth factor 5 levels (GCST90274790), and IL-12 subunit beta levels (GCST90274798) (**Figure 2A**). Thirty-six SNPs in GCST90274765 were strongly associated with thymic malignant tumors. Twenty-nine SNPs in GCST90274773 were strongly associated with thymic malignant tumors. Thirty-three SNPs in GCST90274790 were strongly associated with thymic malignant tumors. Thirty-six SNPs in GCST90274798 were strongly associated with thymic malignant tumors.

Nine SNP datasets were strongly associated with thymic benign tumors, namely, the levels of adenosine deaminase (GCST90274759), caspase 8 (GCST90274763), C-C motif chemokine 28 (GCST90274769), IL-10 receptor subunit beta (GCST90274797), IL-12 subunit beta (GCST90274798), latency-associated peptide transforming growth factor beta 1 (GCST90274818), programmed cell death 1 ligand 1 (GCST90274832), TNF-related apoptosis-inducing ligand (GCST90274843), and TNF-related activation-induced cytokine (GCST90274844) (**Figure 2B**). Twenty-three SNPs in GCST90274759 were strongly associated with thymic benign tumors. Twenty-three SNPs in GCST90274763 were strongly associated with thymic benign tumors. Thirty-six SNPs in GCST90274769 were strongly associated with thymic benign tumors. Twenty-seven SNPs in GCST90274797 were strongly associated with thymic benign tumors. Thirty-six SNPs in GCST90274798 were strongly associated with thymic benign tumors. Thirty SNPs in GCST90274818 were strongly associated with thymic benign tumors. Twenty-six SNPs in GCST90274832 were strongly associated with thymic benign tumors. Thirty-seven SNPs in GCST90274843 were strongly associated with thymic benign tumors. Forty-six SNPs in GCST90274844 were strongly associated with thymic benign tumors. The package “MendelianRandomization” in R was used to calculate the *F*-value of SNPs and remove confounding factors. No weak IVs or confounding factors were found (**Table 2**).

**Table 2:** Cytokine-related GWAS data.

id.exposure	Name	nsnp	Tumor
GCST90274765	C-C motif chemokine 19 levels	36	Malignant neoplasm of the thymus (controls excluding all cancers)
GCST90274773	T-cell surface glycoprotein CD5 levels	29	Malignant neoplasm of the thymus (controls excluding all cancers)
GCST90274790	Fibroblast growth factor 5 levels	33	Malignant neoplasm of the thymus (controls excluding all cancers)
GCST90274798	Interleukin-12 subunit beta levels	36	Malignant neoplasm of thymus (controls excluding all cancers)
GCST90274759	Adenosine deaminase levels	23	Benign neoplasm: Thymus
GCST90274763	Caspase 8 levels	23	Benign neoplasm: Thymus
GCST90274769	C-C motif chemokine 28 levels	36	Benign neoplasm: Thymus
GCST90274797	Interleukin-10 receptor subunit beta levels	27	Benign neoplasm: Thymus
GCST90274798	Interleukin-12 subunit beta levels	36	Benign neoplasm: Thymus
GCST90274818	Latency-associated peptide transforming growth factor beta 1 levels	30	Benign neoplasm: Thymus
GCST90274832	Programmed cell death 1 ligand 1 levels	26	Benign neoplasm: Thymus
GCST90274843	TNF-related apoptosis-inducing ligand levels	37	Benign neoplasm: Thymus
GCST90274844	TNF-related activation-induced cytokine levels	46	Benign neoplasm: Thymus

### 3.2 Results of MR analysis

IVW and MR-Egger regression were used to determine the causal relationship between cytokine expression and thymic benign and malignant tumors. The TwoSampleMR package was used for visualizing the results, including scatter plots and forest plots.

**Figure 3:**
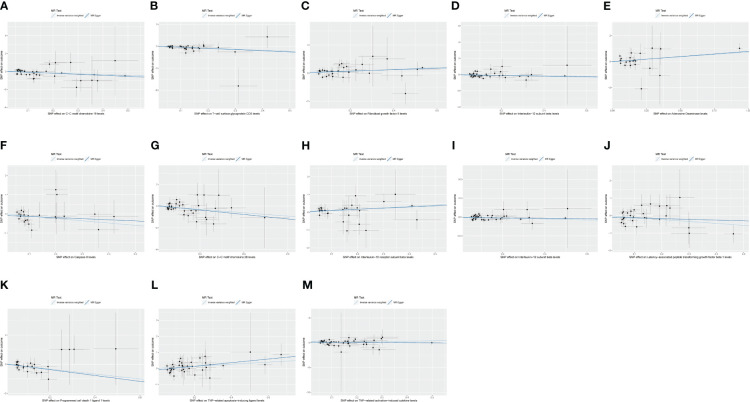
**(A)** Scatter plot showing the causal relationship between C-C motif chemokine 19 levels and malignant neoplasm of thymus (controls excluding all cancers), evaluated by the IVW method. **(B)** Scatter plot showing the causal relationship between T-cell surface glycoprotein CD5 levels and malignant neoplasm of thymus (controls excluding all cancers), evaluated by the IVW method. **(C)** Scatter plot showing the causal relationship between fibroblast growth factor 5 levels and malignant neoplasm of thymus (controls excluding all cancers), evaluated by the IVW method. **(D)** Scatter plot showing the causal relationship between interleukin-12 subunit beta levels and malignant neoplasm of thymus (controls excluding all cancers), evaluated by the IVW method. **(E)** Scatter plot showing the causal relationship between adenosine deaminase levels and benign neoplasm of the thymus evaluated by the IVW method. **(F)** Scatter plot of the causal relationship between caspase 8 levels and benign neoplasm of the thymus, evaluated by the IVW method. **(G)** Scatter plot of the causal relationship between C-C motif chemokine 28 levels and benign neoplasm of the thymus, evaluated by the IVW method. **(H)** Scatter plot of the causal relationship between interleukin-10 receptor subunit beta levels and benign neoplasm of the thymus, evaluated by the IVW method. **(I)** Scatter plot of the causal relationship between interleukin-12 subunit beta levels and benign neoplasm of the thymus, evaluated by the IVW method. **(J)** Scatter plot of the causal relationship between latency-associated peptide transforming growth factor beta 1 levels and benign neoplasm of the thymus, evaluated by the IVW method. **(K)** Scatter plot of the causal relationship between programmed cell death 1 ligand 1 levels and benign neoplasm of the thymus, evaluated by the IVW method. **(L)** Scatter plot of the causal relationship between TNF-related apoptosis-inducing ligand levels and benign neoplasm of the thymus, evaluated by the IVW method. **(M)** Scatter plot of the causal relationship between TNF-related activation-induced cytokine levels and benign neoplasm of the thymus, mainly evaluated by the IVW method.

C-C motif chemokine 19 levels showed a causal relationship with thymic malignant tumors and was a protective factor (IWV, *p* = 0.017, OR = 0.429, 95% CI: 0.214–0.860). There was a causal relationship between T-cell surface glycoprotein CD5 levels and thymic malignant tumors, which was a protective factor (IWV, *p* = 0.0026, OR = 0.251, 95% CI: 0.102–0.618). There was a causal relationship between fibroblast growth factor 5 levels and thymic malignant tumors, which was a risk factor (IWV, *p* = 0.0045, OR = 1.915, 95% CI: 1.224–2.997). There was a causal relationship between IL-12 subunit beta levels and thymic malignancies (IWV, *p* = 0.043, OR = 0.593, 95% CI: 0.358–0.984) (**Figures 3A–D**).

There was a causal relationship between adenosine deaminase levels and thymic benign tumors, a risk factor (IWV, *p* = 0.042, OR = 2.082, 95% CI: 1.026–4.225). There was a causal relationship between caspase 8 levels and thymic benign tumors, a protective factor (IWV, *p* = 0.048, OR = 0.303, 95% CI: 0.093–0.989). C-C motif chemokine 28 levels showed a causal relationship with thymic benign tumors and was a protective factor (IWV, *p* = 0.0068, OR = 0.213, 95% CI: 0.070–0.653). There was a causal relationship between IL-10 receptor subunit beta levels and thymic benign tumors, a risk factor (IWV, *p* = 0.0094, OR = 2.269, 95% CI: 1.223–4.209). There was a causal relationship between IL-12 subunit beta levels and thymic benign tumors, a protective factor (IWV, *p* = 0.029, OR = 0.531, 95% CI: 0.301–0.937). Latency-associated peptide transforming growth factor beta 1 levels showed a causal relationship with thymic benign tumors (IWV, *p* = 0.042, OR = 0.327, 95% CI: 0.111–0.961). A causal relationship existed between programmed cell death 1 ligand 1 level and thymic benign tumors, a protective factor (IWV, *p* = 0.0029, OR = 0.185, 95% CI: 0.061–0.562). TNF-related apoptosis-inducing ligand levels showed a causal relationship with thymic benign tumors, a risk factor (IWV, *p* = 0.049, OR = 1.910, 95% CI: 1.000–3.646). TNF-related activation-induced cytokine levels showed a causal relationship with thymic benign tumors, a risk factor (IWV, *p* = 0.043, OR = 2.187, 95% CI: 1.024–4.673) (**Figures 3E–M**).

**Figure 4:**
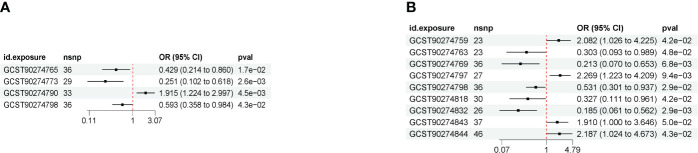
**(A)** Forest map showing the causal results between cytokines and malignant neoplasm of the thymus (controls excluding all cancers) (IVW method). **(B)** Forest map of causal results between cytokines and benign neoplasm of the thymus (IVW method).

The results of MR analysis showed a causal relationship between IL-12 subunit beta levels and thymic malignant tumors, a protective factor. There was a causal relationship between IL-12 subunit beta levels and thymic benign tumors, a protective factor. IL-12 subunit beta was a common cytokine affecting both thymic malignancies and benign tumors (**Figures 4A, B**).

### 3.3 Sensitivity analysis

**Figure 5:**
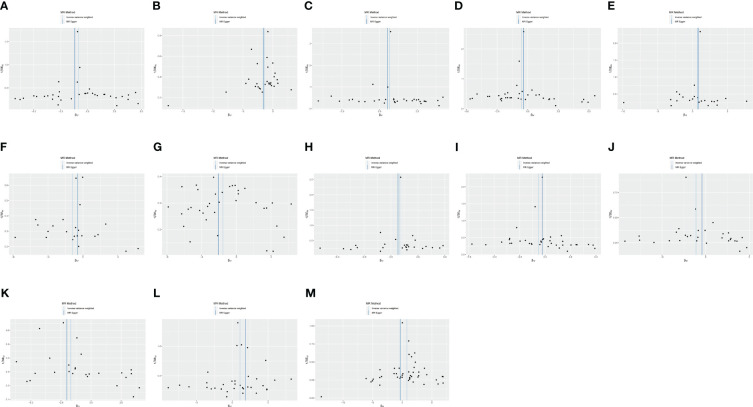
**(A)** Funnel plot of the causal relationship between C-C motif chemokine 19 levels and malignant neoplasm of the thymus (controls excluding all cancers), evaluated by the IVW method. **(B)** Funnel plot of the causal relationship between T-cell surface glycoprotein CD5 levels and malignant neoplasm of the thymus (controls excluding all cancers), evaluated by the IVW method. **(C)** Funnel plot of the causal relationship between fibroblast growth factor 5 levels and malignant neoplasm of thymus (controls excluding all cancers), evaluated by the IVW method. **(D)** Funnel plot of the causal relationship between interleukin-12 subunit beta levels and malignant neoplasm of the thymus (controls excluding all cancers), evaluated by the IVW method. **(E)** Funnel plot of the causal relationship between adenosine deaminase levels and benign neoplasm of the thymus, evaluated by the IVW method. **(F)** Funnel plot of the causal relationship between caspase 8 levels and benign neoplasm of the thymus, evaluated by the IVW method. **(G)** Funnel plot of the causal relationship between C-C motif chemokine 28 levels and benign neoplasm of the thymus, evaluated by the IVW method. **(H)** Funnel plot of the causal relationship between interleukin-10 receptor subunit beta levels and benign neoplasm of the thymus, mainly evaluated by the IVW method. **(I)** Funnel plot of the causal relationship between interleukin-12 subunit beta levels and benign neoplasm of the thymus, mainly evaluated by the IVW method. **(J)** Funnel plot of the causal relationship between latency-associated peptide transforming growth factor beta 1 levels and benign neoplasm of the thymus, evaluated by the IVW method. **(K)** Funnel plot of the causal relationship between programmed cell death 1 ligand 1 levels and benign neoplasm of the thymus, evaluated by the IVW method. **(L)** Funnel plot of the causal relationship between TNF-related apoptosis-inducing ligand levels and benign neoplasm of the thymus, evaluated by the IVW method. **(M)** Funnel plot of the causal relationship between TNF-related activation-induced cytokine levels and benign neoplasm of the thymus, evaluated by the IVW method.

**Figure 6:**
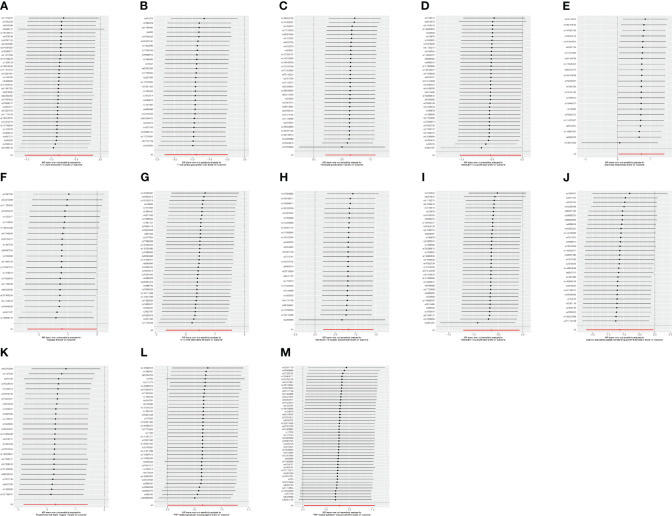
**(A)** “Leave-one-out” plot of the causal relationship between C-C motif chemokine 19 levels and malignant neoplasm of the thymus (controls excluding all cancers), evaluated by the IVW method. **(B)** “Leave-one-out” plot of the causal relationship between T-cell surface glycoprotein CD5 levels and malignant neoplasm of the thymus (controls excluding all cancers), evaluated by the IVW method. **(C)** “Leave-one-out” plot of the causal relationship between fibroblast growth factor 5 levels and malignant neoplasm of the thymus (controls excluding all cancers), evaluated by the IVW method. **(D)** “Leave-one-out” plot of the causal relationship between interleukin-12 subunit beta levels and malignant neoplasm of the thymus (controls excluding all cancers), evaluated by the IVW method. **(E)** “Leave-one-out” plot of the causal relationship between adenosine deaminase levels and benign neoplasm of the thymus, evaluated by the IVW method. **(F)** “Leave-one-out” plot of the causal relationship between caspase 8 levels and benign neoplasm of the thymus, evaluated by the IVW method. **(G)** “Leave-one-out” plot of the causal relationship between C-C motif chemokine 28 levels and benign neoplasm of the thymus, evaluated by the IVW method. **(H)** “Leave-one-out” plot of the causal relationship between interleukin-10 receptor subunit beta levels and benign neoplasm of the thymus, evaluated by the IVW method. **(I)** “Leave-one-out” plot of the causal relationship between interleukin-12 subunit beta levels and benign neoplasm of the thymus, evaluated by the IVW method. **(J)** “Leave-one-out” plot of the causal relationship between latency-associated peptide transforming growth factor beta 1 levels and benign neoplasm of the thymus, evaluated by the IVW method. **(K)** “Leave-one-out” plot of the causal relationship between programmed cell death 1 ligand 1 levels and benign neoplasm of the thymus, evaluated by the IVW method. **(L)** “Leave-one-out” plot of the causal relationship between TNF-related apoptosis-inducing ligand levels and benign neoplasm of the thymus, evaluated by the IVW method. **(M)** “Leave-one-out” plot of the causal relationship between TNF-related activation-induced cytokine levels and benign neoplasm of the thymus, evaluated by the IVW method.

Three methods are often used to evaluate sensitivity. The heterogeneity test assesses the differences between individual IVs. If the differences between different IVs are large, then the heterogeneity among these IVs is large. The pleiotropy test is used to test whether multiple IVs show horizontal pleiotropy. It is commonly expressed by the intercept term of MR-Egger’s method. If the intercept term is far from 0, it indicates horizontal pleiotropy. The leave-one-out sensitivity approach is the MR result of evaluating the remaining IVs after eliminating IVs one by one. If there is a large difference between the MR results estimated using other IVs and the total results after eliminating one IV, it indicates sensitivity to that IV. MR-Egger regression and the IVW method showed a *p* > 0.05 for all datasets with no heterogeneity (**Table 3**). The funnel plot showed a symmetric distribution of SNPs, and the obtained results were relatively stable (**Figure 5**). MR-Egger regression analysis was used to test for the presence of directional horizontal pleiotropy. A *p* > 0.05 for all datasets indicated no horizontal pleiotropy. The leave-one-out method was used to eliminate SNPs one by one and observe the change in the effect size. No SNPs with a strong influence were found (**Figure 6**).

**Table 3:** Heterogeneity analysis.

id.exposure	Name	Heterogeneity tests		Directional horizontal pleiotropy tests
		MR-Egger	IVW	
GCST90274765	C-C motif chemokine 19 levels	0.656	0.671	0.427
GCST90274773	T-cell surface glycoprotein CD5 levels	0.931	0.947	0.828
GCST90274790	Fibroblast growth factor 5 levels	0.817	0.836	0.529
GCST90274798	Interleukin-12 subunit beta levels	0.758	0.78	0.549
GCST90274759	Adenosine deaminase levels	0.293	0.344	0.818
GCST90274763	Caspase 8 levels	0.777	0.802	0.537
GCST90274769	C-C motif chemokine 28 levels	0.416	0.453	0.638
GCST90274797	Interleukin-10 receptor subunit beta levels	0.876	0.895	0.602
GCST90274798	Interleukin-12 subunit beta levels	0.676	0.670	0.307
GCST90274818	Latency-associated peptide transforming growth factor beta 1 levels	0.265	0.276	0.408
GCST90274832	Programmed cell death 1 ligand 1 levels	0.759	0.800	0.788
GCST90274843	TNF-related apoptosis-inducing ligand levels	0.938	0.927	0.256
GCST90274844	TNF-related activation-induced cytokine levels	0.862	0.801	0.099

## 4 Discussion

The present study used GWAS data on 91 cytokines as IVs to identify potential causal cytokines in thymic malignancies and benign tumors using MR analysis. There was a causal relationship between fibroblast growth factor 5 levels and thymic malignancies and it was a risk factor. C-C motif chemokine 19 levels, T-cell surface glycoprotein CD5 levels, and IL-12 subunit beta levels were causally related to thymic malignant tumors. These were protective factors. Adenosine deaminase levels, IL-10 receptor subunit beta levels, TNF-related apoptosis-inducing ligand levels, and TNF-related activation-induced cytokine levels exhibited a causal relationship with thymic benign tumors and were risk factors. Caspase 8 levels, C-C motif chemokine 28 levels, IL-12 subunit beta levels, latency-associated peptide transforming growth factor beta 1 levels, and programmed cell death 1 ligand 1 levels showed a causal relationship with thymic benign tumors, which were protective factors. IL-12 subunit beta was a common cytokine affecting both thymic malignant and benign tumors and was a protective factor for both tumors.

Thymoma is a less malignant and aggressive tumor. Owing to the variety of tumor subtypes and the lack of unique morphological and immunological characteristics, progress in thymoma-related studies is relatively slow (33, 34). However, molecular mechanistic and clinical research on thymoma has progressed with the development of imaging diagnosis technology, surgical methods, systemic therapy, and radiotherapy technology (35). IL-12 is a cytokine comprising two subunits, of which the β subunit is the IL-12 subunit beta. IL-12 is an important immune regulator (36). It regulates and mobilizes the immune system. IL-12 comprises two subunits: p35 and P40; these form an active dimer of IL-12 through a noncovalent linkage. Among them, the P40 subunit is IL-12 subunit beta. IL-12 is secreted by immune cells, including activated macrophages, dendritic cells, and B lymphocytes (37). It is important in promoting Th1 cell response, enhancing NK cell activity, and enhancing cell-mediated immune response. IL-12 subunit beta has a crucial functional role in the cytokine IL-12, vital for regulating the immune system and immune responses (38, 39).

Research on IL-12β in thymic tumors is scanty but some studies have shown that IL-12β may be involved in the occurrence and development of thymic tumors. Some experimental studies showed that IL-12β participates in regulating the immune system. It has a crucial role in controlling tumor development (40). IL-12β can promote the differentiation and activation of Th1 cells, enhance the killing activity of NK cells, and promote the activation and effector function of CD8+T cells to inhibit the growth and spread of tumors (41). The expression of IL-12β in patients with thymic tumors may change, suggesting its involvement in the occurrence and development of thymic tumors. However, the specific mechanism of IL-12β action in thymic tumors remains to be explored. Future research can explore the function of IL-12β expression in thymic tumors and its relationship with clinical characteristics. The mechanism of regulation of the tumor immune microenvironment and anti-tumor immune response is expected to contribute to the further understanding of IL-12β‘s mechanism of action in thymic tumors and can provide new targets and strategies for the treatment of thymic tumors (42).

The strengths of this study are as follows: (1) using MR is a rigorous approach to assess causality; (2) large GWAS datasets were utilized, which increased the statistical power; (3) systematic approaches were used to filter SNPs, account for LD, and remove weak/pleiotropic IVs; and (4) appropriate sensitivity analyses were conducted to test assumptions and robustness of the results.

This study has some limitations that need to be addressed: (1) MR-Egger regression and the IVW method showed *p* > 0.05, suggesting no heterogeneity. However, some heterogeneity may arise from the difference in analysis platform, experimental procedure, population, and other IVs. These sources of heterogeneity must be assessed. (2) This study mainly focused on the European population, and there are certain limitations due to the lack of GWAS data from Asian, African, and other populations. (3) The number of IVs of SNPs in each dataset of this study is not large; thus, large sample data are needed to select more SNPs as IVs for further analysis. (4) In this study, the causal relationship between cytokine expression and benign and malignant thymic tumors was analyzed by MR. The potential causal cytokines related to benign and malignant thymic tumor pathogenesis were identified, but the underlying mechanism was not studied.

## 5 Conclusion

In summary, cytokines showed a causal relationship with benign and malignant thymic tumors. IL-12 subunit beta is a common cytokine affecting malignant and benign thymic tumors, and is a protective factor for both tumors.

## Data availability statement

The datasets presented in this study can be found in online repositories. The names of the repository/repositories and accession number(s) can be found below: https://storage.googleapis.com/finngen-public-data-r10/summary_stats/finngen_R10_C3_THYMUS_EXALLC.gz and https://storage.googleapis.com/finngen-public-data-r10/summary_stats/finngen_R10_CD2_benign_thymir.gz.

## Author contributions

KZ: Data curation, Formal analysis, Methodology, Software, Supervision, Writing – original draft. JS: Conceptualization, Investigation, Software, Writing – original draft. RY: Data curation, Methodology, Project administration, Resources, Writing – review & editing. CZ: Conceptualization, Data curation, Validation, Writing – original draft. ZL: Funding acquisition, Resources, Writing – review & editing.
